# Diminishing personal information privacy weakens image concerns

**DOI:** 10.1371/journal.pone.0232037

**Published:** 2020-04-27

**Authors:** Yohanes E. Riyanto, Jianlin Zhang

**Affiliations:** 1 Division of Economics, School of Social Sciences, Nanyang Technological University, Singapore, Singapore; 2 Academic Division, Singapore Institute of Management, Singapore, Singapore; 3 Department of Economics, Faculty of Arts and Social Sciences, National University of Singapore, Singapore, Singapore; Rutgers The State University of New Jersey, UNITED STATES

## Abstract

The popularity of social media has increased users’ social visibility. However, users’ limited ability to control information spread could compromise privacy. People care about how others perceive them. We examined people’s concerns for others’ evaluations on their behaviors under different degrees of privacy conditions. Using a variant of the dictator game, we induced dictators to self-select into pro-self or pro-social types and asked recipients to give written evaluations of the dictators. We varied the degree of personal information privacy by making the written content known to the corresponding dictators only, all dictators, or either of them with equal chance. Also, the dictators could avoid receiving the message at a price. We showed that pro-self dictators’ willingness to pay to conceal messages decreased when information privacy diminished. Thus, results indicated that image concerns wane in an environment where information privacy is weak. Our results contribute to understanding of the privacy paradox.

## Introduction

People care about the way others perceive them and aspire to be seen as fair, altruistic, and trustworthy [1–8]. Image concerns have been one of the primary motivations for a wide range of behaviors, including voting, education choice, charitable giving, etc. [9]. Other people’s evaluation of oneself is also important in the formation of one’s self-image, as people’s self-appraisal is, to a significant extent, derived from their interpretation of others’ evaluations of them [10, 11]. Because this reflected appraisal process shapes the way people see themselves, it affects their judgment about others’ views of them and, consequently, their relationships with others as well as a host of socioeconomic decisions [12].

Recent advances in information technology and its applications in social media have empowered users with excellent connectivity [13, 14]. However, this ease of social connectivity comes at a potential cost. Users’ personal information could become visible to many others with whom users have no intention of connecting. Indeed, such limited control over the spread of personal information has dramatically compromised user privacy and caused serious concerns among users and regulators. This research focuses on answering an important question of whether a link between the loss of personal information privacy and people’s image concerns exists. Specifically, we examine people’s concerns for others’ evaluations and judgments on their behaviors under different degrees of information privacy conditions. The latter is captured by the likelihood that the contents of others’ evaluations are made known to anonymous third parties in a double-blind setting, where the identities of all involved parties are unknown to each other. In the current social media age, where people and information are intricately linked, answers to this inquiry are of utmost importance. To the best of our knowledge, this paper is the first that investigates this research question.

To this end, we designed and conducted a two-stage laboratory experiment. In stage one of the experiment, we elicited participants’ image concerns based on a dictator game with *ex-post* feedback. In stage two, we conducted a Becker-DeGroot-Marschak (BDM) incentive-compatible bidding mechanism [15] that measures the strength of participants’ image concerns under different degrees of information privacy. As mentioned earlier, our study is closely related to the privacy paradox [16–20]. Existing literature on the privacy paradox is predominantly based on surveys that elicit users’ stated preferences towards the maintenance of information privacy. In contrast, our experimental design allows us to elicit the revealed preference about attitude towards information privacy, which complements well the stated preference approach [21].

Specifically, in stage one, each of the dictators was asked to choose one of two available allocation options: ($7, $7) or ($10, $2). The former allocation choice is fair and collectively efficient, while the latter is unfair and collectively inefficient, as it gives a disproportionally higher payoff to the dictator at the expense of the recipient a lower aggreagate payoffs. Before the dictator decided on the allocation, the dictator was told the recipient had been informed of the two available allocation choices and the respective payoff consequences to both parties. Anticipating that the recipient also knew the link between the allocation choices and payoff outcomes would imply that the dictator could have envisioned the recipient’s image of the dictator, just as the dictator was about to make the allocation choice [22].

Once the allocation decision was made, the dictator was informed that the recipient would write a message of personal opinion about the dictator. Because the dictator’s identity was anonymous to the recipient, the dictator’s image is hence solely dependent on the allocation choice made by the dictator. To illustrate, a choice of ($7, $7) would project an image of a friendly, fair, and altruistic dictator. Expecting that the recipient could potentially express a positive opinion about the dictator would elicit the dictator’s feeling of pride. Conversely, a choice of the unfair and pro-self allocation could disappoint the recipient and sour the recipient’s evaluation of the dictator. An expectation of receiving the recipient’s disapproval could then trigger a sense of moral failure and, consequently, arouse an emotional feeling of shame or guilt [23, 24].

Research in social psychology shows that the general tendency of an ashamed person in the short term is to hide or withdraw from situations that trigger the shameful feelings to protect further image damage [23–26]. As such, an opportunity to take such a course of action was given to the dictator in stage two of the experiment. Specifically, the dictator could pay to stop the message from being revealed through a Becker-DeGroot-Marschak (BDM) incentive-compatible bidding mechanism [15]. Notably, the payoffs resulting from allocation decisions would not change, whether the dictator chose to read the recipient’s message or not. We define a pro-self dictator as a dictator who chooses ($10, $2), which is the unfair, pro-self and collectively inefficient allocation, instead of ($7, $7), which is the fair, pro-social and collectively efficient allocation. If a pro-self dictator pays to avoid knowing others’ opinions of her, the willingness to pay to conceal the expected negative evaluation thus measures her motive to hide or withdraw from the other party’s disapproval and captures the degree of care about her image persona in the other party’s eyes [27].

We varied the degree of personal information privacy in three experimental treatments. In one treatment—which we labelled the *Private* treatment—the dictators were told prior to the bidding process that by default, their respective recipient’s written feedback would be passed on to them. In this treatment, the privacy of the message content was fully protected, as only the dictators and their corresponding recipients would know about the message content. If the dictators did not want to receive the message, they could submit a bid to prevent the message from reaching them. However, the written feedback would be passed on to them if their bids were unsuccessful.

In another treatment—which we labelled the *Public* treatment—the dictators were told that by default, the message contents would be shown and read aloud to all dictators and experimenters in the room. Even though the identity of the dictators remained anonymous, the public disclosure of the message content created an awareness in the targeted dictator’s mind that the recipient’s evaluation would no longer be private, unless the dictator succeeded in concealing the message through the bidding process. The third treatment—which we labelled the *Partially Private* treatment—was similar to the public treatment except that the message is publicly disclosed with a 50% chance and only privately disclosed with a 50% chance in case of an unsuccessful bid. In it, the dictators were told if the bid was unsuccessful, the message would have an equal chance of either being disclosed publicly, or only in private to the target dictator.

Across the three treatments, the dictators thus faced different levels of information privacy about the recipient’s opinion of the dictator. Results indicated that when communication between the two parties was exchanged privately on a one-to-one basis, the pro-self dictators revealed significant concerns about their expectedly negative image. Even though knowledge of the recipient’s opinions did not change the monetary payoffs resulting from allocation decisions, the pro-self dictators were willing to spend significant private resources to avoid knowing the recipient’s opinions of them. Similar to many online communications, in the *Public* treatment, contents of the written messages from recipients were shown to others publicly. Would exposure of the message content heighten the pro-self dictator’s image concerns? We hypothesized two possible effects: the *social norm effect* and the *shame/ guilt sharing effect*. Thus, we predicted the presence of the two effects would reduce the pro-self dictator’s image concerns in the face of weakened information privacy.

Past research shows that the presence of others who are unconcerned about their own privacy reduces one’s privacy concerns; specifically, because people are often uncertain about their privacy preferences, available cues are hence important in guiding their decisions regarding information divulgence [19]. One important cue that people consider involves others’ information disclosure decisions. Treating this as a guide, people often reciprocate by divulging their private information to a similar extent [28], even to computer agents who provide information about themselves [29]. In short, merely observing other people revealing information increases the likelihood that people will reveal their private information to others [30].

In the *Public* treatment, the message content was, by default, unprotected and would be disclosed to all others. For a dictator, such an information divulgence setting could raise awareness that the messages written to other dictators would also be disclosed simultaneously. More generally, if the lack of personal information privacy is a feature of social institutional design, the dictator is just one of many others affected by exactly the same extent. Hence, letting go of her message is in line with the social norm and the “right” choice to make in this environment. We call this behavior the *social norm effect*.

Second, research in social psychology has long demonstrated that social influence and shared stimuli sway people’s decision-making process [31, 32]. In the area of consumer behavior, the findings illustrate that consumers’ consumption experiences are significantly enhanced as soon as they realize that others also develop similar experiences from the same hedonic consumptions. In line with the idea that “*happiness shared is doubled*, *and sadness shared is halved*,” this enhancement takes place regardless of whether the consumer’s initial consumption experience is enjoyable or dreary [33, 34]. Indirect extrapolations from the ideas give rise to the *shame/guilt sharing effect*. Specifically, the awareness that messages written to many other pro-self dictators would also be disclosed at the same time could provide an excuse for pro-self dictators to share their shame or guilt feelings with other pro-self dictators, who are expected to be equally guilty or shameful. This would further reduce the psychological discomfort the pro-self dictators would experience and, consequently, the impetus to withdraw from the situation.

Even though the dictator’s identity is kept anonymous, effectively, the message written by the recipient serves as an evaluation of the dictator’s behavior. Knowledge of the message contents could affect the dictator’s self-appraisal thereby heightening his or her self-image concerns [10, 11]. The presence of the two effects–the *social norm effect* and the *shame/ guilt sharing effect*–would, however, reduce the image concerns. We thus hypothesized that the degree of image concerns is lower in the *Public* treatment than in the *Private* treatment.

## Materials and methods

### Participants

A total of 374 undergraduate students of the Singapore Institute of Management—majoring in business studies, information technology, and arts and social sciences—participated. The experiment was conducted using pen and paper. The participants did not know one another prior to the experiment, and none had previously participated in similar experiments. The study was approved by the ethics committee of the Singapore Institute of Management. Participants were financially compensated for their participation. The study was incentivized, did not involve deception and thus met the standards in psychology and behavioral economic research.

### General experimental procedure

On arrival at the experiment site, participants were randomly assigned the role of either dictator or recipient. Depending on their role, they were then directed to two separate, adjacent rooms. The number of participants in each room was the same. The experimenters then read aloud the instructions to the participants and verified they all understood the game, with any query answered individually in private.

The experiment commenced only when all participants understood the game. The experiment was comprised of two stages. Before stage one began, dictators were informed that they would be asked to make two decisions, with one decision being randomly chosen by the experimenters to calculate the dictator’s earnings from the experiment. However, dictators were also informed that the nature of the second decision would be revealed only after all dictators made the first decision.

### Stage one: Allocation decision

**Fig 1:**
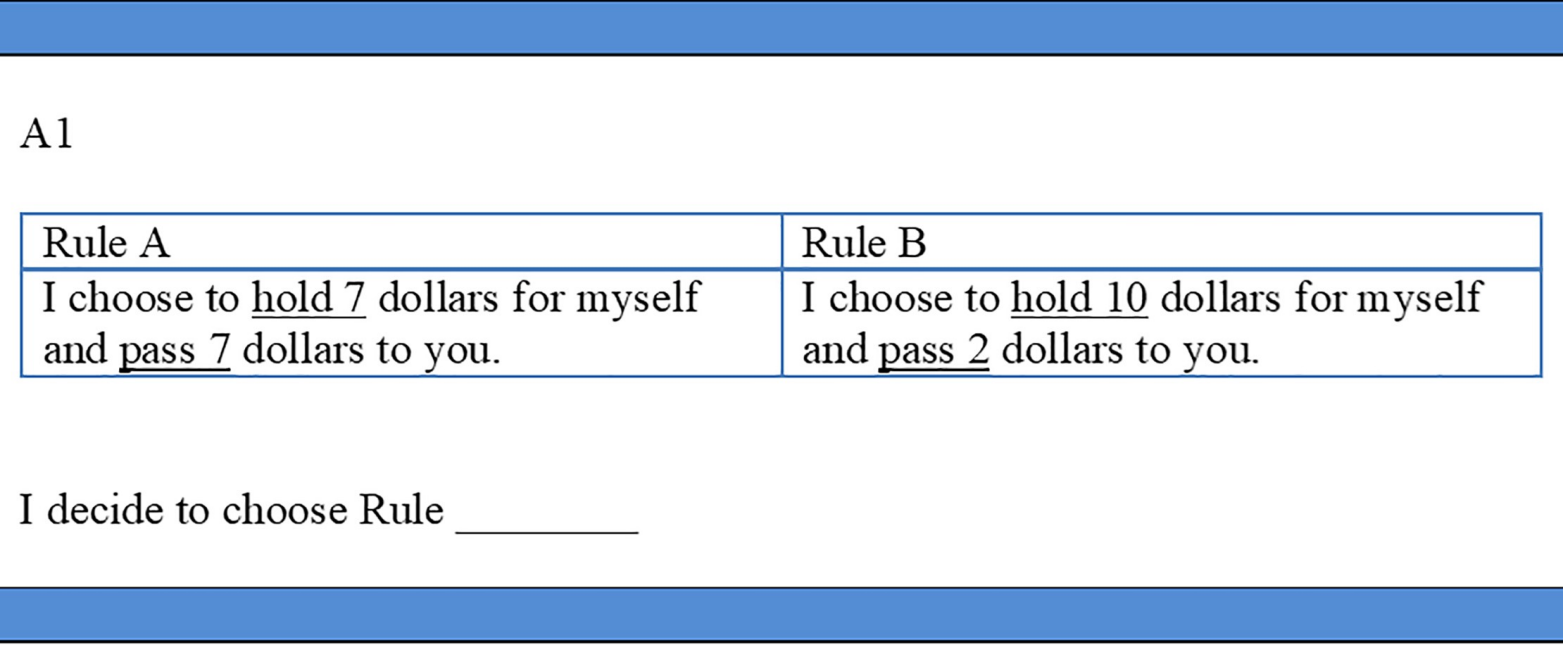
Allocation decision sheet. This figure shows the stage one decision sheet for dictators, who were asked to choose one rule from the two shown on the decision sheet. Before making the stage one decision, dictators were also told that the matched recipients had been informed about the two decision rules for splitting the money.

At the start of stage one, dictators were asked to choose one of two options—“I choose to hold 7 dollars for myself and pass 7 dollars to you” or “I choose to hold 10 dollars for myself and pass 2 dollars to you”—for splitting the money (in Singapore dollars) between themselves and a matched recipient. Dictators were also told that matched recipients were informed about the game rules and dictators’ two options for splitting the money. Once all dictators understood the game, they privately indicated their chosen allocation decisions on an allocation decision sheet shown in Fig 1. Dictators were then asked to insert the decision sheet into an envelope and drop the envelope into a box in the room’s corner. Both the decision sheet and envelope were marked with the dictator’s identity number. Each participant knew that no one, except himself or herself, knew about his or her unique identity number. Hence, neither the experimenters nor other participants would be able to link the identity number with his or her name or face.

After all dictators made their allocation decisions, the experimenters collected the envelopes and placed them in plain view in the room’s corner. Payments to the recipients (based on allocation decisions), together with the dictators’ allocation decision sheets were inserted into their respective envelopes. These envelopes were then collected and placed in a box, which was taken to the recipients’ rooms. In addition, dictators could observe the experimenters taking the box to the rooms. Each recipient was then asked to pick one envelope at random, verify that the monetary amount matched the allocation decision, and take the money. Thereafter, recipients were asked to write a short message (on the back of the allocation decision sheet) stating their personal opinion of the relevant dictator. Other than a prohibition against threatening or foul language, no restriction was imposed on message content. Once recipients wrote the message, they inserted it into the same envelope and dropped it back into the box for experimenters to collect and return to the dictators’ rooms. This completed stage one of the experiment.

### Stage two: Bidding decision

In stage two, the dictators were told that their matched recipients had received payment according to their chosen allocation option. In addition, each recipient had written a short message expressing a personal opinion of the dictator. Each dictator was then asked to make a second decision: they were required to state the maximum amount they were willing to pay (through a BDM mechanism) to stop the written message from being passed on to the dictator.

Notably, the experimental instructions for dictators were divided into two parts. The first part contains the general instructions that explain how dictators should make their allocation decisions in stage one. The second part describes the BDM mechanism, employed in the bidding decision in stage two. Furthermore, these instructions were given to the dictators only after completion of stage one. Thus, no foreknowledge existed that there would be feedback from the recipient and that this feedback might be made public—so this had no influence on allocation decisions. Separation of instructions into two parts was needed to prevent the dictators from behaving strategically by changing their allocation decision, knowing that the recipients were going to give their written opinion of the dictators.

**Fig 2:**
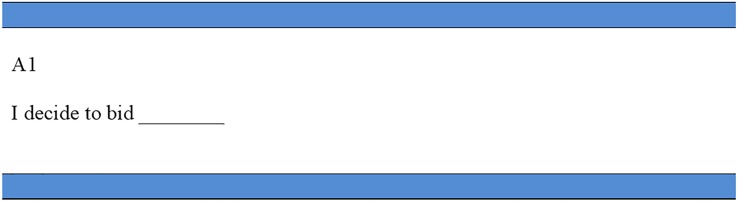
Bidding decision sheet. This figure shows the decision sheet for the BDM procedure. Dictators were given $10 to participate in the bidding process. Dictators were asked to indicate an amount they were willing to pay to conceal the message. Permitted bids ranged from $0 to $10, in one-dollar increments.

Each dictator was given $10 to participate in the bidding process. Care was taken to ensure that all participants understood the procedures of the BDM mechanism before the bidding process started. Through the BDM mechanism, we first asked dictators to indicate on a bidding decision sheet the amount they were willing to pay to conceal the messages. The decision sheet shown in Fig 2 was marked with the dictator’s identity number. The permitted bids ranged from $0 to $10, in one-dollar increments. Once all dictators decided on their bids, they dropped their decision sheets into a box located in the room’s corner, for experimenters to collect. Using the identity numbers, experimenters then put each bidding decision sheet into the corresponding dictator’s envelope.

Once all dictators submitted their bids, experimenters then drew a random price—ranging from $1 to $10—using a random number generator. If the bid submitted by a dictator was larger than or equal to the random price, the bid was considered successful, and the message written to the dictator was removed from the envelope; thus, the dictator would not know the message content. Earnings from stage two equaled the difference between the $10 endowment and the random price. If the submitted bid was less than the random price, the bid was considered unsuccessful, and the dictator’s earnings from stage two equaled the $10 endowment. Additionally, the dictator would be informed of the message content.

### Treatment manipulation

**Fig 3:**
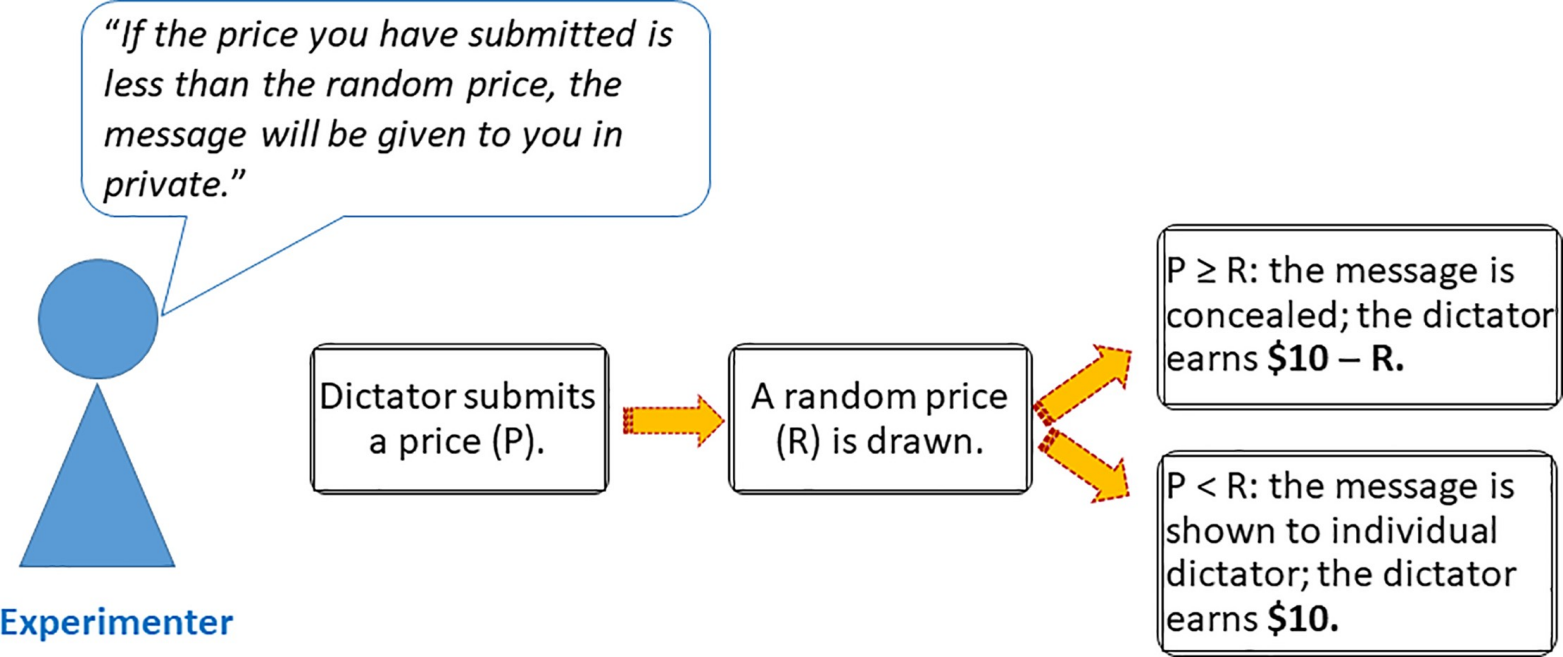
The BDM procedure in the *Private* treatment. Before the bidding started, the experimenter informed dictators that an unsuccessful bid would result in dictators privately receiving messages written to them. When the bidding started, dictators first submitted a price. Thereafter, the experimenter drew a random price using a random number generator. The experimenter then compared the submitted price to the random price. The bid was successful if the submitted price was greater or equal to the random price, and unsuccessful otherwise.

**Fig 4:**
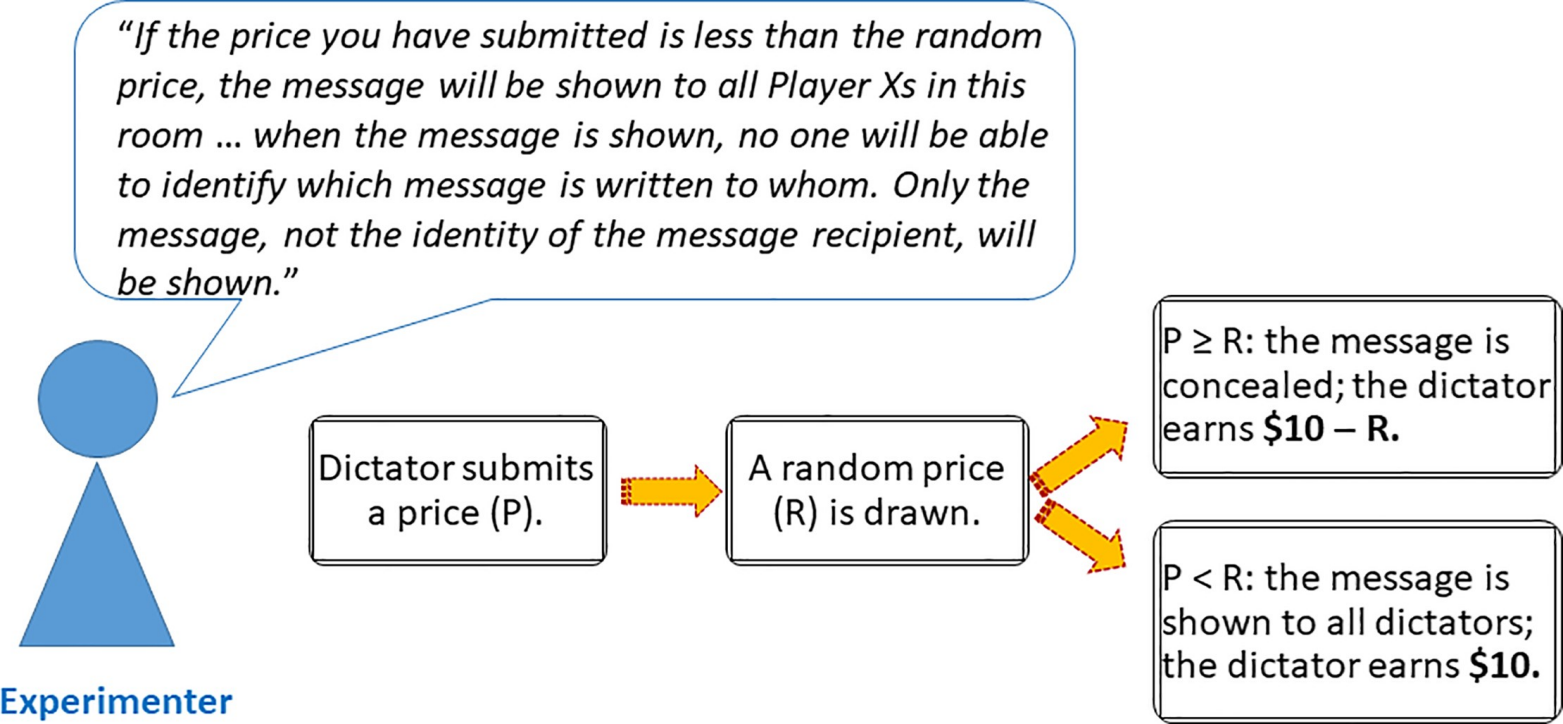
The BDM procedure in the *Public* treatment. Before the bidding started, the experimenter informed dictators that an unsuccessful bid would result in messages written to them being shown to all dictators. However, when the message was shown to all dictators, the identity of the dictator would be preserved. When the bidding started, the dictator first submitted a price. Thereafter, the experimenter drew a random price using a random number generator. The experimenter then compared the submitted price to the random price. The bid was successful if the submitted price was greater or equal to the random price, and unsuccessful otherwise.

**Fig 5:**
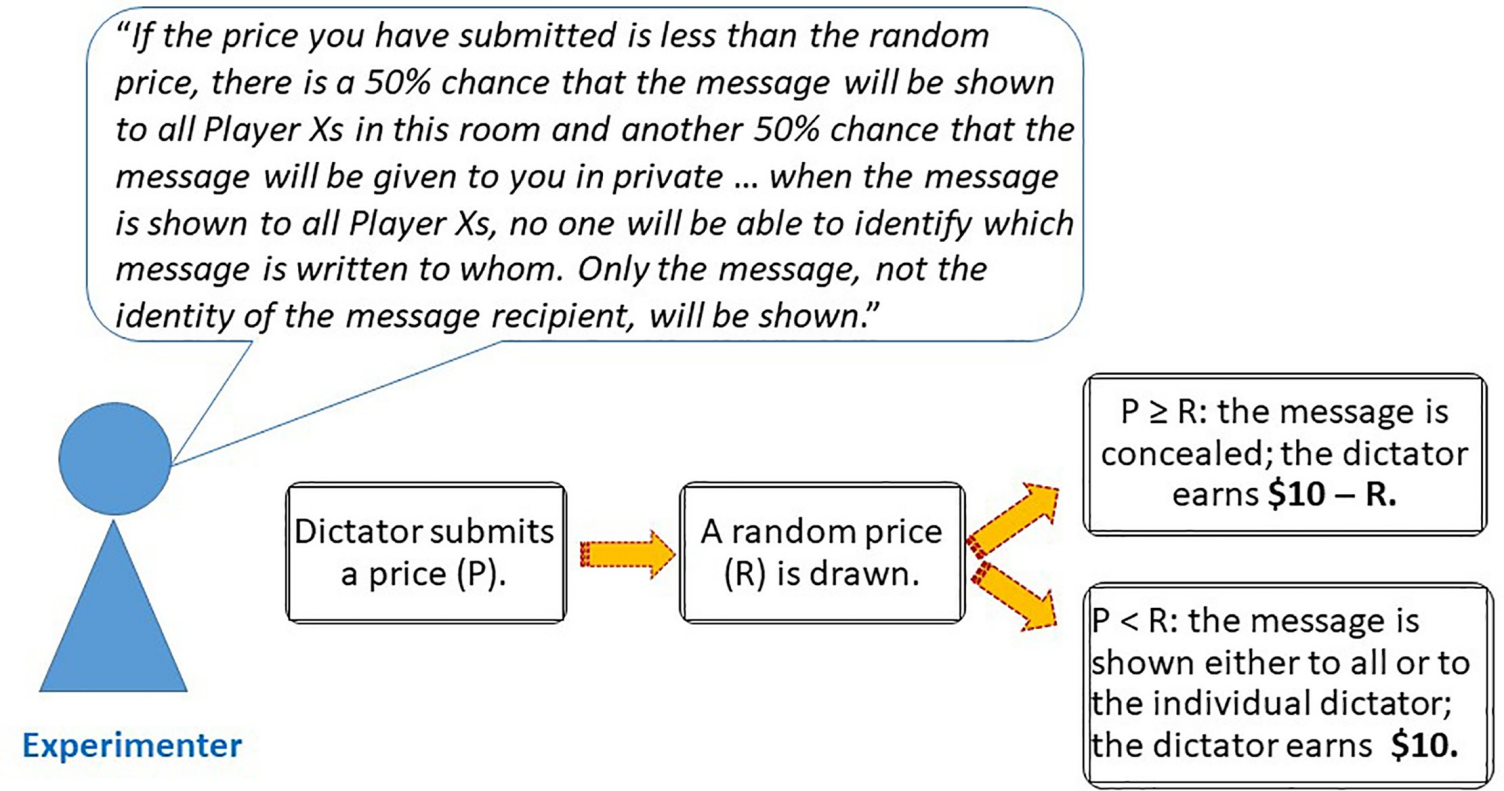
The BDM procedure in the *Partially Private* treatment. Before the bidding started, the experimenter informed dictators that an unsuccessful bid would result in either dictators privately receiving the message written to them, or the message written to them being shown to all dictators. However, when the message was shown to all dictators, the identity of the dictator would be preserved. When the bidding started, the dictator first submitted a price. Thereafter, the experimenter drew a random price using a random number generator. The experimenter then compared the submitted price to the random price. The bid was successful if the submitted price was greater or equal to the random price, and unsuccessful otherwise.

We implemented three experimental treatments: the *Private* treatment, the *Public* treatment, and the *Partially Private* treatment. The treatments differed in the way messages were shown to dictators whose bids were unsuccessful. The dictators were given clear information about the way recipients’ messages would be revealed. Graphical illustrations for the BDM mechanism in each of the treatments are shown in Figs 3, 4 and 5.

In the *Private* treatment, messages for dictators whose bids were unsuccessful were inserted into the respective dictators’ envelopes. In the *Public* treatment, messages for dictators whose bids were unsuccessful were placed on a visualizer projector and read aloud to all participants and experimenters in the room. However, except for the targeted dictator, no one knew for whom the message was intended. In the *Partially Private* treatment, half the messages for dictators whose bids were unsuccessful were randomly selected and inserted into the respective dictators’ envelopes. The other half were shown on a visualizer projector and read aloud to all participants and experimenters. For detailed information, see the instructions for the experiment (S1 File). At the experiment’s end, all envelopes, including those of dictators whose bids were successful, were then placed inside a box located in the room’s corner. Subsequently, the dictators were asked to collect their envelopes (distinguished by dictator identity number).

Immediately after the bidding process was completed, experimenters randomly chose one of the two decisions—from the allocation and bidding stages—to determine dictators’ final earnings. According to the decisions made, a monetary payment to each dictator was then inserted into his/her envelope. All dictators were asked to collect their own envelopes (distinguished by dictator identity number). Before dictators were allowed to leave the room, they were asked to stay a few minutes, in order to check if monetary payments were correct and to read any messages. Dictators then took the payments and left the experiment’s site.

Overall, we conducted eight experimental sessions. Three sessions were conducted for the *Private* treatment, three for the *Public* treatment, and two for the *Partially Private* treatment. Treatments and sessions were conducted in random order, with each session lasting approximately 45 to 50 minutes. Participants’ final earnings ranged from $7 to $15, including the $5 attendance fee. The exchange rate for 1 Singapore Dollar is about 75 US Cents and the hourly wage for campus job in Singapore is around 8 Singapore Dollars at the time when experiment was conducted. About 80% of participants who assumed the role of dictator were Singaporean students; the rest were from other Asian countries, such as Malaysia and Indonesia. Participants did not know one another prior to the experiment, and none had previously participated in similar experiments.

## Results

Table 1 summarizes the descriptive statistics. The proportion of subjects who self-selected into the unfair and socially inefficient allocation option ranged from 35% to 42% across the three treatments.

**Table 1:** Summary of descriptive statistics.

		Private		Partially Private		Public	
Number of allocators		62		47		77	
Number of recipients		62		47		77	
Total		124		94		154	
Gender of allocators	Male	19 (30.65%)		20 (42.55%)		28 (36.36%)	
	Female	43 (69.35%)		27 (57.45%)		49 (63.64%)	
Allocation	Option	($7, $7)	($10, $2)	($7, $7)	($10, $2)	($7, $7)	($10, $2)
	No. Obs	37 (59.68%)	25 (40.32%)	27 (57.45%)	20 (42.55%)	50 (64.94%)	27 (35.06%)
Bid	Average	1.27	3.28	1.52	2.65	1.26	1.56
	Median	0.00	3.00	0.00	2.50	0.00	1.00
	Std. Dev.	1.90	2.74	2.19	2.18	2.23	1.97

In this table, we provide a breakdown of the number of participants, their roles, and their gender in the three treatments. We also summarize the dictators’ allocation decisions.

Two observations are worth making. First, upon knowing that the message was going to be revealed to all dictators, the pro-self dictators generally exhibited a stronger desire to conceal the message, except when the message was going to be shown publicly. Across the three treatments, the bid amounts submitted by the pro-self dictators to conceal messages were larger than those of the pro-social dictators. Throughout the paper, we report the *p-value* from the Mann-Whitney *(MW)* test for the equality of the means of bid amounts between a pair of treatments. The difference between the bid amounts by the two types of dictators is statistically significant at the 1% significance level in the *Private* treatment (*MW*, *p-value* = 0.002) and at the 5% significance level in the *Partially Private* treatment (*MW*, *p-value* = 0.047). However, the difference is not significant in the *Public* treatment (*MW*, *p-value* = 0.193). The fact that the pro-self dictators are always willing to pay higher amounts in the *Private* treatment may show their inclination to conceal the message.

**Fig 6:**
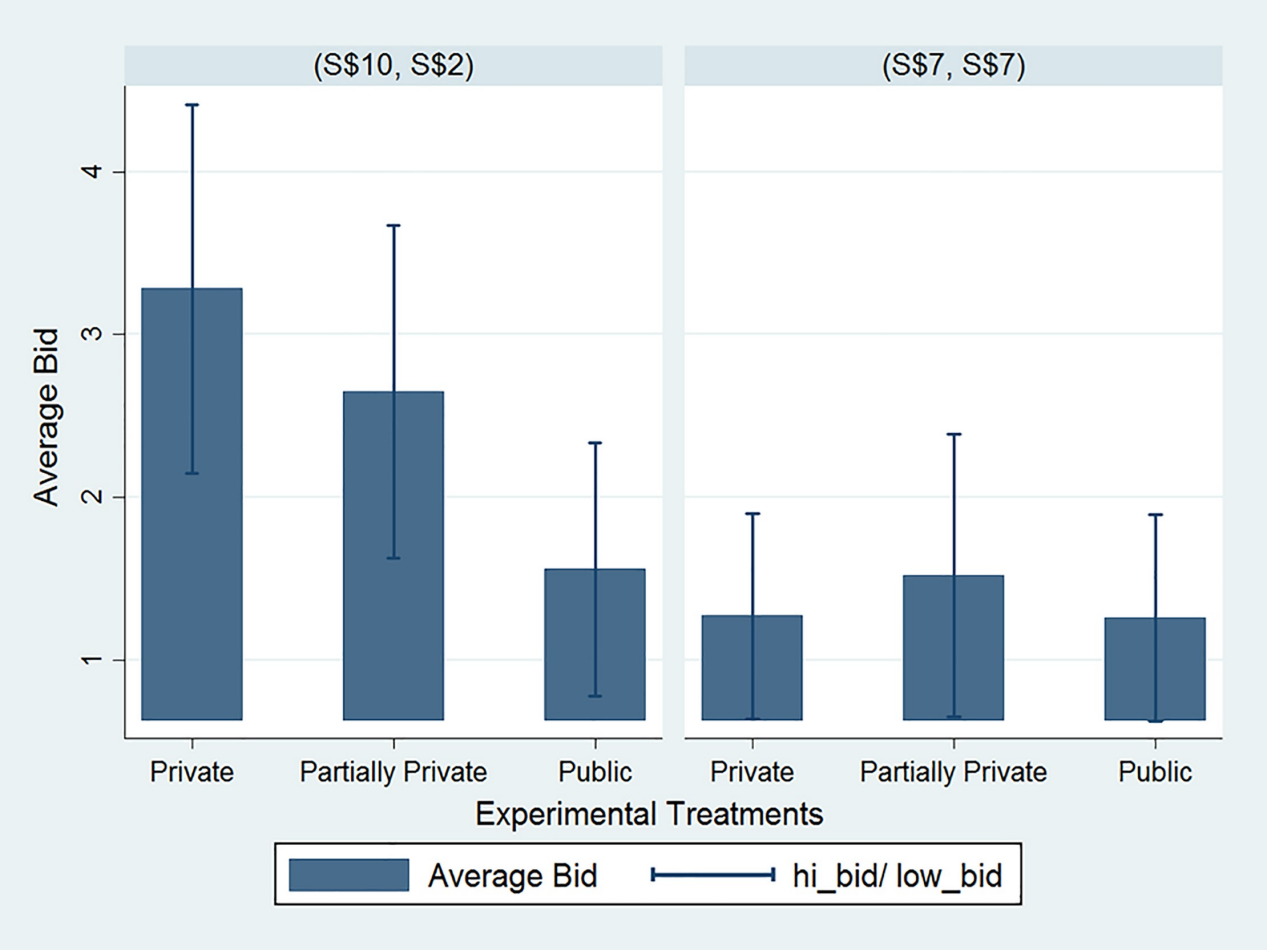
Average bids across treatments. This figure shows average bids exerted by dictators to conceal messages across three treatments, *Private*, *Partially Private*, and *Public* treatments, with varying degrees of information privacy. The left panel is for dictators who chose the unequal allocation (S$10; S$2), and the right panel is for dictators who chose the equal allocation (S$7; S$7). Standard error bars are shown.

Second, with regard to the pro-self and socially inefficient allocation, the bid amounts tended to decrease as the probability of the message being shown publicly to passive others increased. This suggests that pro-self dictators exhibited less willingness to conceal messages when faced with increasing probability of losing protection on the message content privacy in the event of an unsuccessful bid. However, the change in privacy protection did not exert a significant impact on the pro-social dictators’ willingness to conceal messages. The bid amounts made by pro-social dictators were comparably low across the three treatments. These findings are evident in Fig 6, which conveys the average bid amounts across the three treatments by both types of dictators.

With regard to the pro-self and socially inefficient allocation, there was no significant difference between the average bid amounts by the pro-self dictators in the *Private* and *Partially Private* treatments (*MW*, *p-value =* 0.5172). The difference between the average bid amounts in the *Partially Private* and *Public* treatments is weakly statistically significant (*MW*, *p-value =* 0.0700). When we compared the bid amounts in the *Private* and *Public* treatments, we found the difference statistically significant (*MW*, *p-value =* 0.0181).

In contrast, when the pro-social allocation was chosen, the bid amounts did not differ much across treatments. The Mann-Whitney test results indicate the difference is not statistically significant (*MW*, *p-value* = 0.7217, *p-value =* 0.392, and *p-value =* 0.584, respectively). Given that the expected perceptions of the dictators were likely to be positive in case of the fair allocation, there should have been no strong urge to conceal the anticipated positive message, regardless of whether the message was shown privately to the dictator or publicly to all dictators and experimenters. One possible factor behind the decision of some pro-social dictators to conceal an expected positive message could be their sense of modesty. That is, they would prefer to stay behind the veil of ignorance. In a companion paper [27], we show that the pro-social dictators adopted a neutral attitude towards others’ expectedly fair perception of them. The willingness to pay to reveal the recipient’s message was low when they were allowed to do so privately. Would the pro-social dictators bid higher amounts if a successful bid results in the message shown to others as in the *Public* treatment? The findings could be relevant to literature on social approval and disapproval as rewarding and punishment mechanism.

To consider other variables that might affect results, we also present a series of simple ordinary least squares (OLS) regressions on the pro-self dictators’ bid amounts, representing the dependent variable. The independent variables are *D_Private*, an indicator variable taking the value of 1 if the subject was in the *Private* treatment and 0 otherwise; *D_PartiallyPrivate*, an indicator variable with the value of 1 if the subject was in the *Partially Private* treatment and 0 otherwise; and *Gender*, an indicator variable with the value of 1 if the subject was female and 0 otherwise. Table 2 summarizes the regression results, which further confirm our previous observations.

**Table 2:** OLS regression results.

	(1)	(2)
VARIABLES	Bid Amount	Bid Amount
D_Private	1.630**	1.630**
	(0.663)	(0.669)
D_Partial_Private	1.044	1.043
	(0.667)	(0.669)
Gender	0.734	0.727
	(0.636)	(0.660)
Nationality		-0.0309
		(0.561)
Session	-0.0426	-0.0411
	(0.355)	(0.363)
Constant	1.152	1.173
	(0.884)	(0.925)
Observations	72	72
R-squared	0.115	0.115

Robust standard errors in parentheses

*** p<0.01

** p<0.05

* p<0.1

Results of the OLS bid amount regressions by dictators who chose the ($10, $2) allocation. *D_Private* and *D_Partially_Private* denote the *Private* and *Partially Private* treatments, respectively. Robust standard errors are in parentheses. ** denotes significance at the 5% level, and * denotes significance at the 10% level.

As Table 2 illustrates, the difference in *Private* and *Public* treatment bid amounts is statistically significant (*p-value*<0.05). The bid amount in the *Private* treatment was, on average, around $1.63 higher than in the *Public* treatment. The bid amount in the *Partially Private* treatment was, on average, around $1.04 higher than in the *Public* treatment; however this difference in bid amounts is not statistically significant. Overall, OLS regression results suggest that the possibility of having the recipients’ messages of the pro-self and unfair allocation shared with the audience blunted the dictators’ incentive to conceal the message. Note that the difference in the *Partially Private* and *Private* treatment bid amounts is not statistically significant (*p-value* = 0.44). Neither the *Gender* variable nor the *Session* variable is statistically significant in the regression results, suggesting that gender did not affect pro-self dictators’ bid amounts and there is no session effect.

We also examined the content of some messages sent by recipients to both pro-self and pro-social dictators. In general, messages written to pro-self dictators were negative. For example, one recipient said to a selfish dictator, “*Wow*! *You are selfish*. *If you don’t feel you are selfish*, *then you are worse than selfish*.” Another said, “*Money is the root of all evil*, *or greed*. *(You) should at least divide it equally to be fair*. *Oh well*, *GREEDY*.” In contrast, messages sent to fair-minded dictators were generally pleasant, such as “*You are a good person*” and “*May God bless you*!”

## Discussion

Similar to communication in an offline context, the privacy of the message content was fully protected in the *Private* treatment. For a pro-self dictator, reading the personally written message alone was akin to facing the recipient and being rebuked in person for the committed moral failure. The thought of such an experience would induce feelings of shame or guilt. Therefore, the dictator would naturally want to avoid such feelings. In this way, the dictators’ willingness to pay to conceal the message is a good measure of their image concerns.

In the *Public* treatment and, to a lesser extent, the *Partially Private* treatment, a pro-self dictator knew that sharing the message content was a feature of institutional design that affected all dictators. Furthermore, blame on the dictator would be disclosed together with the blame on many other pro-self dictators. The recognition that a particular dictator was just one of many dictators whose committed moral failures would be exposed publicly thus provided an opportunity to share the dictator’s shame or guilt feelings, hence weakening the dictator’s desire to hide from knowing the expectedly negative image of the dictator. All in all, our results showed that a loss of information privacy weakened image concerns.

Our results could potentially contribute to the understanding of privacy paradox [16–21]. People often claim that control over personal data is essential to them. Yet, by and large, they also remain casual about relinquishing the control. This dichotomy in the attitude adopted towards privacy and behaviors has long puzzled researchers [35, 36]. The behaviors outlined in the privacy paradox are widely observed in social media. Absence of privacy risk awareness among users, as well as users’ lack of understanding of ways to protect privacy were found to be a major cause of the paradox [37–40].

We argue that discrepancy between users’ attitudes towards privacy and their online practice could be a product of the online social environment. In stark contrast to off-line communication, which offers deliberate connectivity and measured privacy protection, socializing on networking platforms offers a quick way to fill people’s innate desire to be socially connected with others. With the click of a mouse, users can instantly connect with others, whose personal information then becomes readily available at the user’s disposal. Prolonged exposure to an environment characterized by widespread sharing of personal information could cultivate a reciprocal behavior from users in the form of sharing their personal information too. This could potentially foster a mutual understanding that sharing of personal information is part and parcel of the online social norm, easing users’ privacy concerns whenever exposing themselves to the online environment.

Making information public has been found to increases image concerns and, thereby, fostering desired behaviors in a wide range of activities. Using a revised public good game, it was shown that personal investments in climate protection increased substantially if players can invest publicly and gains social reputation [41]. In the area of pro-environmental behavior, for example, it was shown that consumers exhibit a greater willingness to pay for more expensive green products if the buying decision is public [42, 43]. These findings seemingly contradict the result in the current study. One possible solution to this seeming contradiction is that the interaction in the current study is strictly one-shot. Since subsequent interaction as part of the experiment does not exist, reputational building through demonstration of pro-social behaviors cannot be part of player’s strategy. This renders public display of message contents irrelevant. The removal of reputational building motive is further strengthened by the double-blind experimental design implemented in the experiment. The design guarantees that no one, except the dictator himself or herself, knows his or her decisions made during or after the experiment. Further, no one, except the target dictator, knows which message is written to him or her. This common knowledge of anonymity in decision maker’s identity thus makes reputation building a futile endeavor which could explain why image concerns waned in the face of decreasing information privacy [44].

Interaction in the Private treatment is akin to one-to-one communication between the dictator and the recipient. Suppose the pro-self dictator was an introvert individual, he or she would be inclined to avoid communicating with the recipient in such a setting as introvert individuals tend to be more apprehensive in face-to-face communication [45–47]. However, there was no difference in the bid amounts from the pro-social dictators in the *Private* treatment and those in the *Public* treatment. The bid amounts were low in both treatments suggesting that pro-social dictators in the *Private* treatment did not show a different tendency to avoid communicating with the recipients. To ascertain potential impacts that personal characteristics might exert, it might be necessary to study players’ response to a neutral decision without injecting shame or guilt feeling in stage one, rather than the dictator game in the present study. Explanation based on communication avoidance would be strengthened further if participants in the *Private* treatment based on the neutral decision still show a strong tendency to escape the one-to-one like communication.

Our design ensures complete anonymity of the decisions. That is, except the dictator himself or herself, no one, including the experimenters, can identify which dictator made what decisions *during* and *after* the experiment. Even though the proximity between any two dictators was at least two seats apart in our experimental setup, using a paper-and-pencil design could, nevertheless, result in a weaker feeling of anonymity among the dictators than that based on online-interaction [48]. Further, the fact that dictators were able to see other dictators in the same room might potentially heighten the dictator’s image concerns [49]. To strengthen external validity, in future research it would be interesting to investigate if behaviors are consistent in an online environment with a strengthened anonymity elicited by physically segregated dictators.

## Supporting information

S1 File Full version of experimental instructions. (DOCX)

S1 Data (DO)

S2 Data (DTA)

## References

[1] Andreoni J, Petrie R. Public goods experiments without confidentiality: A glimpse into fund-raising. Journal of Public Economics 2004; 88(7–8): 1605–1623.

[2] Ariely D, Bracha A, Meier S. Doing good or doing well? Image motivation and monetary incentives in behaving prosocially. American Economic Review 2009; 99(1): 544–555.

[3] Bénabou R, Tirole J. Incentives and prosocial behavior. American Economic Review 2006; 96(5): 1652–1678.

[4] Bénabou R, Tirole J. Laws and norms 2012. IZA Discuss. Pap. 6290: 1–44.

[5] Ellingsen T, Johannesson M. Pride and prejudice: The human side of incentive theory. American Economic Review 2008; 98(3): 990–1008.

[6] Andreoni J, Bernheim BD. Social image and the 50–50 norm: A theoretical and experimental analysis of audience effects. Econometrica 2009; 77(5): 1607–1636.

[7] Bursztyn L, Jensen R. How does peer pressure afect educational investments? Quartly Journal of Economics 2015; 130(3): 1329–1367. 10.1093/qje/qjv021 PMC4698889 26740727

[8] Rodriguez Mosquera PM, Fischer AH, Manstead ASR, Zaalberg R. Attack, disapproval, or withdrawal? The role of honour in anger and shame responses to being insulted. Cognition and Emotion 2008; 22(8): 1471–1498.

[9] Bursztyn L, Jensen R. Social image and economic behavior in the field: Identifying, understanding and shaping social pressure. Annual Review of Economics 2016; 9: 131–153.

[10] Cooley CH. Human Nature and the Social Order. New York: Schocken; 1964.

[11] Shaffer L. From mirror self-recognition to the looking-glass self: Exploring the justification hypothesis. Journal of Clinical Psychology 2005; 61(1): 47–65. 10.1002/jclp.20090 15558625

[12] Wallace HM, Tice DM. Reflected appraisal through a 21st-century looking glass. In Leary MR, Tangney JP. eds. Handbook of Self and Identity, Vol.2 New York: Guilford; 2012 pp. 124–140.

[13] Ellison NB, Steinfield C, Lampe C. The benefits of Facebook ‘‘friends”: Social capital and college students’ use of online social network sites. Journal of Computer-Mediated Communication 2007; 12(4): 1143–1168.

[14] Stefanone MA, Jang CY. Writing for friends and family: The interpersonal nature of blogs. Journal of Computer-Mediated Communication 2008; 13(1): 123–140.

[15] Becker G, DeGroot M, Marschak J. Measuring utility by a single-response sequential method. Behavioral Sciences 1964; 9(3): 226–236. 10.1002/bs.3830090304 5888778

[16] Barnes S. A privacy paradox: Social networking in the United States. First Monday 2006; 11(9): 4 September.

[17] Gross R, Acquisti A. Information revelation and privacy in online social networks (The Facebook case). ACM workshop on privacy in the electronic society; 2005 11 07–07; Alexandria, VA, USA https://dl.acm.org/citation.cfm?id=1102214.

[18] Acquisti A, Gross R. Predicting social security numbers from public data. Proceedings of the National Academy of Sciences 2009; 106(27): 10,975–10,980. 10.1073/pnas.0904891106 PMC2706270 19581585

[19] Acquisti A, Brandimarte L, Loewenstein G. Privacy and human behavior in the age of information. Science 2015; 347: 509–514. 10.1126/science.aaa1465 25635091

[20] Adjerid I, Acquisti A, Brandimarte L, Loewenstein G. Sleights of privacy: Framing, disclosures and the limits of transparency. Symposium on usable privacy and security (SOUPS); 2013 7 24–26; Newcastle, UK.; Article No. 9.

[21] Athey S, Catalini C, Tucker C. The digital privacy paradox: Small money, small costs, small talk 2017; https://papers.ssrn.com/sol3/papers.cfm?abstract_id=2916489.

[22] Xiao E, Houser D. Avoiding the sharp tongue: Anticipated written messages promote fair economic exchange. Journal of Economic Psychology 2009; 30(3): 393–404.

[23] Scheff TJ. Shame and the social bond: A sociological theory. Sociological Theory 2000; 18: 84–99.

[24] Keltner D, Harker LA. The forms and functions of nonverbal signal of shame. In Gilbert P, Andrews B, eds. Shame: Interpersonal Behavior, Psychopathology, and Culture New York, NY: Oxford University Press; 1998 pp. 78–98.

[25] Lewis M. Shame, the Exposed Self. New York, NY: Free Press; 1992.

[26] Tangney JP, Dearing RL. Shame and Guilt. New York, NY: Guilford; 2002.

[27] Riyanto YE, Zhang J. Putting a price tag on others' perception of us. Experimental Economics 2016; 19(2): 480–499.

[28] Derlega VJ, Chaikin AL. Privacy and self-disclosure in social relationships. Journal of Social Issues 1977; 33(3):102–115.

[29] Moon Y. Intimate exchanges: Using computers to elicit self-disclosure from consumers. Journal of Consumer Research 2000; 26(4): 323–339.

[30] Acquisti A, John LK, Loewenstein G. The impact of relative standards on the propensity to disclose. Journal of Marketing Research 2012; 49(2): 160–174.

[31] Asch SE. Opinions and Social Pressure. Scientific American 1955; 193(5): 31–35.

[32] Deutsch M, Gerard HB. A Study of normative and informational social influences and individual judgment? Journal of Abnormal and Social Psychology 1955; 51(3): 629–36. 10.1037/h0046408 13286010

[33] Raghunathan R, Corfman K. Is happiness shared doubled and sadness shared halved? Social influence on enjoyment of hedonic experiences. Journal of Marketing Research 2006; 43(3): 386–94.

[34] Ramanathan S, McGill A. Consuming with others: Social influences on moment-to-moment and retrospective evaluations of an experience. Journal of Consumer Research 2007; 34(4): 506–524.

[35] Robert SL, Maxine W. Privacy as a concept and a social issue: A multidimensional developmental theory. Journal of Social Issues 1977; 33(3): 22–42.

[36] Acquisti A. Privacy in electronic commerce and the economics of immediate gratification. Proceedings of the 5th ACM conference on Electronic commerce; 2004 May 17–20; New York, NY, USA. pp. 21–29.

[37] Acquisti A, Gross R. Awareness, information sharing, and privacy on the Facebook. Paper presented at Privacy Enhancing Technologies, 6th International Workshop; 2006 June 28–30; Cambridge, UK.

[38] Boyd D, Hargittai E. Facebook privacy settings: Who cares? First Monday 2011; 15(8).

[39] Debatin B, Lovejoy JP, Horn AK, Hughes BN. Facebook and online privacy: Attitudes, behaviors, and unintended consequences. Journal of Computer-Mediated Communication 2009; 15(1): 83–108.

[40] Tufekci Z. Can you see me now? Audience and disclosure regulation in online social network sites. Bulletin of Science, Technology & Society 2008; 28(1): 20–36.

[41] Milinski M, Semmann D, Krambeck HJ, Marotzke J. Stabilizing the Earth’s climate is not a losing game: Supporting evidence from public goods experiments. Proceedings of the National Academy of Sciences 2006; 103(11): 3994–3998. 10.1073/pnas.0504902103 PMC1449634 16537474

[42] Berger J. Signaling can increase consumers' willingness to pay for green products. Theoretical model and experimental evidence. Journal of Consumer Behaviour 2019; 18: 233–246.

[43] Berger J. Are luxury brand labels and “green” labels costly signals of social status? An extended replication. PlOS ONE 2017; 10.1371/journal.pone.0170216 PMC5295666 28170399

[44] Gambetta D, Przepiorka W. Natural and strategic generosity as signals of trustworthiness. PlOS ONE 2014; 10.1371/journal.pone.0097533 PMC4022519 24831097

[45] Scott C. Preference for online social interaction: A theory of problematic internet use and psychosocial well-being. Communication Research 2003;30(6):625–648

[46] Werner S, Nguyen A, Durkin K. Shyness and computer-mediated communication: A self-presentational theory perspective. Media Psychology 2004; 6(1):1–22.

[47] Hammick JK, Lee M. Do shy people feel less communication apprehension online? The effects of virtual reality on the relationship between personality characteristics and communication outcomes. Computers in Human Behavior 2014; 33:302–310.

[48] Franzen P. Anonymity in the dictator game revisited. Journal of Economic Behavior & Organization 2012; (81), 74–81.

[49] Bateson M, Nettle D, Roberts G. Cues of being watched enhance cooperation in a real-world setting. Biology Letters 2006; 2(3): 412–414. 10.1098/rsbl.2006.0509 17148417 PMC1686213

