# Supplementary material for: Diminishing personal information privacy weakens image concerns
Source: PLoS One. 2020 Apr 27;15(4):e0232037. doi: 10.1371/journal.pone.0232037 (PMC7185703; doi:10.1371/journal.pone.0232037)
Supplement: S1 File — (DOCX) [file pone.0232037.s001.docx]

General Instruction

This is an experiment in decision-making. By participating in this experiment, all participants, including the participants in the next room, have earned a $5 participation fee. You will also earn additional payments from one of the two decisions that you will be asked to make. The exact amount you will be paid will depend on the decisions you make. At the end of the second decision, one decision will be randomly chosen to calculate your final payments. You do not know which decision will be chosen. Both are equally likely to be chosen. As such, you should treat both decisions seriously.

At the end of the experiment, we will insert your payment inside a sealed envelope. You will pick the envelope in a designated place. No one other than you yourself will ever know how much you have earned from this experiment.

To preserve your anonymity, each of you is given a unique identity number. You will be identified only by your identity number throughout the whole experiment. Neither the experimenter nor the other participants will know your personal identity during and after the study has been completed. We assure you that your personal identity and the decision you make will be kept completely private. All information collected will strictly be kept confidential for the sole purpose of this study.

Throughout the experiment, please do not talk or try to communicate with other participants during the experiment. Please put away all outside materials (such as book bags, notebooks) before starting the experiment. If you have a question during the experiment, raise your hand, and an experimenter will assist you. Participants violating the rules will be asked to leave the experiment and will not be paid.

Instruction for Decision One (all treatments)

In this experiment, each of you is paired with one other participant in the next room. The grouping will be anonymous and random. No one will ever know who your matched participant is in the next room. Your matched participant in the next room will never know who you are.

All participants will be randomly assigned a role in this experiment. All participants in this room are Player X. All participants in the other room are Player Y. Thus, the number of Player Ys in the other room is exactly the same as the number of Player Xs in this room. The difference between these roles is described below.

You (Player X) are given two dividing rules to divide money between you and your matched Player Y. A dividing rule determines how much money you (Player X) will earn in Decision One and how much money will go to your matched Player Y. The payment to your matched Player Y will be solely based on the dividing rule you choose. Your matched Player Y is informed about these two dividing rules. You (Player X) will need to choose ONE of the two rules. The two rules are described below.

| Rule A | Rule B |
| --- | --- |
| You (Player X) choose to hold 7 Dollars for yourself and pass 7 Dollars to your matched Player Y. | You (Player X) choose to hold 10 Dollars for yourself and pass 2 Dollars to your matched Player Y. |

You should indicate your choice on the decision sheet. After you have chosen the rule, please insert the decision sheet into the envelope and drop it into the open box in the corner of the room. No one will ever know which envelope is your envelope.

Instruction for Decision Two (Private Treatment)

Your decision sheet has been passed to your matched Player Y. Your matched Player Y has also received the monetary amount according to the dividing rule you have chosen. After receiving the monetary payment, your matched Player Y is asked to write a message to you. The message is about his or her personal opinion of you based on the dividing rule you have chosen.

By default, you will receive the message written by your matched Player Y in private.

You are given $10 to make Decision Two. You are free to use any amount out of the $10 to make this decision. In this decision, you can pay a price to STOP the message from passing to you. There are 11 prices available ranging from 0 dollar to 10 dollars at 1-dollar increments. Specifically, the available prices are $0, $1, $2, $3, $4, $5, $6, $7, $8, $9, and $10. You should bid (choose) one price from these 11 prices.

Once the experimenter receives all the prices submitted by all Player Xs, the experimenter will randomly choose a price from $1 to $10. The chosen price is called the random price.

If the price you have submitted is Greater than or Equal to the random price, the message written by your matched Player Y WILL NOT be given to you. You will be required to pay the random price. Your earnings from Decision Two will then be 10 dollars minus the random price.

However, if the price you have submitted is Less than the random price, the message will be given to you in private. Only you will know the content of the message. Your earnings from Decision Two will be 10 dollars.

Note that a bid price of $0 means that you will be certain to receive the message written by your matched Player Y because the random price is drawn from $1 to $10. Moreover, if your bid price is higher than the random price, you will be required to pay only the amount equal to the random price. Therefore, you will not lose additional money from submitting a bid that exceeds the random price. The best strategy is to bid exactly what you feel the opportunity to hide the message from you is worth to you.

Instruction for Decision Two (Partial Private Treatment)

Your decision sheet has been passed to your matched Player Y. Your matched Player Y has also received the monetary amount according to the dividing rule you have chosen. After receiving the monetary payment, your matched Player Y is asked to write a message to you. The message is about his or her personal opinion of you based on the dividing rule you have chosen.

By default, there is 50% chance that the message written by your matched Player Y will be shown on the projector to all Player Xs in this room and another 50% chance that the message will be passed to you in private.

You are given $10 to make Decision Two. You are free to use any amount out of the $10 to make this decision. In this decision, you can pay a price to STOP the message from being shown to all Player Xs in this room or passing to you in private. There are 11 prices available ranging from 0 dollar to 10 dollars at 1-dollar increments. Specifically, the available prices are $0, $1, $2, $3, $4, $5, $6, $7, $8, $9, and $10. You should bid (choose) one price from these 11 prices.

Once the experimenter receives all the prices submitted by all Player Xs, the experimenter will randomly choose a price from $1 to $10. The chosen price is called the random price.

If the price you have submitted is Greater or Equal to the random price, the message written by your matched Player Y WILL NOT be shown to any player Xs including you. You will be required to pay the random price. Your earnings from Decision Two will then be 10 Dollars minus the random price.

However, if the price you have submitted is Less than the random price, there is a 50% chance that the message WILL BE SHOWN to all Player Xs in this room and another 50% chance that the message WILL BE passed to you in private. Your earnings from Decision Two will be 10 Dollars. When the message is shown to all Player Xs, no one will be able to identify which message is written to whom. Only the message, not the identity of the message recipient, will be shown.

Note that a bid price of $0 means that the message written by your matched Player Y will definitely be shown to all Player Xs or be given to you in private because the random price is drawn from $1 to $10. Moreover, if your bid price is higher than the random price, you will be required to pay only the amount equal to the random price. Therefore, you will not lose additional money from submitting a bid that exceeds the random price. The best strategy is to bid exactly what you feel the opportunity to hide the message from you is worth to you.

Instruction for Decision Two (Public Treatment)

Your decision sheet has been passed to your matched Player Y. Your matched Player Y has also received the monetary amount according to the dividing rule you have chosen. After receiving the monetary payment, your matched Player Y is asked to write a message to you. The message is about his or her personal opinion of you based on the dividing rule you have chosen.

By default, the message written by your matched Player Y will be shown on the projector to all Player Xs in this room.

You are given $10 to make Decision Two. You are free to use any amount out of the $10 to make this decision. In this decision, you can pay a price to STOP the message from being shown to all Player Xs. There are 11 prices available ranging from 0 dollar to 10 dollars at 1-dollar increments. Specifically, the available prices are $0, $1, $2, $3, $4, $5, $6, $7, $8, $9, and $10. You should bid (choose) one price from these 11 prices.

Once the experimenter receives all the prices submitted by all Player Xs, the experimenter will randomly choose a price from $1 to $10. The chosen price is called the random price.

If the price you have submitted is Greater or Equal to the random price, the message written by your matched Player Y WILL NOT be shown to any Player Xs including you. You will be required to pay the random price. Your earnings from Decision Two will then be 10 Dollars minus the random price.

However, if the price you have submitted is Less than the random price, the message WILL BE SHOWN to all Player Xs in this room. Your earnings from Decision Two will be 10 Dollars. When the message is shown to all Player Xs, no one will be able to identify which message is written to whom. Only the message, not the identity of the message recipient, will be shown.

Note that a bid price of $0 means that the message written by your matched Player Y will definitely be shown to all Player Xs in this room because the random price is drawn from $1 to $10. Moreover, if your bid price is higher than the random price, you will be required to pay only the amount equal to the random price. Therefore, you will not lose additional money from submitting a bid that exceeds the random price. The best strategy is to bid exactly what you feel the opportunity to hide the message from you is worth to you.

Experimental Instruction (Player Y)

This is an experiment in decision-making. By participating in this experiment, all participants, including the participants in the next room, have earned a $5 participation fee.

In this experiment, each of you is paired with one other participant in the next room. The grouping will be anonymous and random. No one will ever know who your matched participant is in the next room. Your matched participant in the next room will never know who you are. All participants will be randomly assigned a role in this experiment.

All participants in this room are Player Y. All participants in the other room are Player X. Thus, the number of Player Xs in the other room is exactly the same as the number of Player Ys in this room. The difference between these roles is described below.

Your matched Player X is asked to divide money between himself (herself) and you. There are two rules to divide the money. Your matched Player X will need to choose ONE from these two rules. Your additional earnings and your matched Player X’s additional earnings will be based on the rule that he (she) chooses. The two rules are described below.

| Rule A | Rule B |
| --- | --- |
| Your matched Player X chooses to hold 7 Dollars for him(her)self and pass 7 Dollars to you. | Your matched Player X chooses to hold 10 Dollars for him (her)self and pass 2 Dollars to you. |

This is how we calculate your payments. If your matched Player X chooses Rule A, you will receive 7 dollars of additional payment. Your total payments will be 12 dollars for this experiment. If your matched Player X chooses Rule B, you will receive 2 dollars of additional payment. Your total payments will be 7 dollars for this experiment.

After your matched Player X has made the decision, the experimenter will prepare your payments according to the rule your matched Player X has chosen. Your payments together with your matched Player X’s decision sheet will be inserted inside an envelope. They will be passed to you in private. You can verify your payments against the decision sheet.

After receiving your payment, you will need to write a message about your personal opinion of your matched Player X. Depending on the further decision of your matched Player X, your message may or may not be shown to your matched Player X. Other than rude (foul) and threatening messages, you can write anything about your matched Player X. There is no restriction on the content of the message, except those rude (foul) and threatening messages.

Once you have written the message, please pocket your payments. Thereafter, please insert your message back into the same envelope and drop it into the open box in the corner of the room. Neither the experimenter nor the other participants will know who writes what message to his (her) matched Player X.
